# Diabetes Mellitus as a Risk Factor for Complicated Urinary Tract Infections in Kidney Transplant Recipients

**DOI:** 10.3390/jcm14020618

**Published:** 2025-01-18

**Authors:** Severins Krohmals, Christophe de Terwangne, Arnaud Devresse, Eric Goffin, Tom Darius, Antoine Buemi, Michel Mourad, Hector Rodriguez-Villalobos, Nada Kanaan

**Affiliations:** 1 Department of Nephrology, Cliniques Universitaires Saint-Luc, Université Catholique de Louvain, 1200 Brussels, Belgium; severinskrohmals@gmail.com (S.K.); arnaud.devresse@uclouvain.be (A.D.); eric.goffin@uclouvain.be (E.G.); 2 Department of Internal Medecine, Cliniques Universitaires Saint-Luc, 1200 Brussels, Belgium; christophe.deterwangne@saintluc.uclouvain.be; 3 Institut de Recherche Experimentale et Clinique (IREC), Université Catholique de Louvain, 1200 Brussels, Belgium; tom.darius@uclouvain.be (T.D.); antoine.buemi@uclouvain.be (A.B.); michel.mourad@uclouvain.be (M.M.); hector.rodriguez@saintluc.uclouvain.be (H.R.-V.); 4 Surgery and Abdominal Transplant Unit, Cliniques Universitaires Saint-Luc, Université Catholique de Louvain, 1200 Brussels, Belgium; 5 Microbiology, Cliniques Universitaires Saint-Luc, Université Catholique de Louvain, 1200 Brussels, Belgium

**Keywords:** urinary infections, diabetes, post-transplant diabetes, risk factors, double J catheter

## Abstract

**Background:** Urinary tract infections (UTIs) are a common complication after kidney transplantation. The aim of this study was to evaluate the impact of pre-existing diabetes mellitus and post-transplant diabetes mellitus (PTDM) on the occurrence of pyelonephritis in kidney transplant recipients. **Methods:** We performed a retrospective analysis which included 299 adult patients transplanted with a kidney between 2018 and 2022. Patients were categorized into pre-transplantation diabetics, PTDM, and non-diabetics. Asymptomatic bacteriuria and lower urinary infections were not included. **Results:** During a median follow-up time of 31 [17–45] months, 100 UTIs were reported in the total cohort, with a mean time from transplantation to the first UTI episode of 10 ± 11 months. At 48 months, the cumulative incidence of UTIs was 34.9%, 56%, and 47.3% for patients without prior diabetes, pre-transplant diabetes, and PTDM, respectively. Pre-transplant diabetes was independently associated with 79% increased risk of UTIs (adjusted HR = 1.79, 95% CI = 1.14–2.81, *p* = 0.011). The risk associated with female gender increased to 85%. Patient survival was not significantly affected by the interaction between diabetes and UTI occurrence. **Conclusions:** Pre-transplant diabetes arises as a significant risk factor for UTIs after kidney transplantation.

## 1. Introduction

Urinary tract infections (UTIs) are among the most common infections following kidney transplantation [1]. Risk factors related to the development of UTIs in kidney transplant recipients (KTR) include female gender, older age, dose and duration of immunosuppression, urological maneuvers, surgical factors such as vesicoureteral reflux, and co-morbidities such as diabetes mellitus [2]. Within the first year post-transplantation, complicated UTIs frequently present as pyelonephritis or bacteremia, markedly elevating the risk of kidney allograft dysfunction and recurrent bacterial infections [3], particularly in patients with diabetes mellitus. Moreover, the microbiological landscape of UTIs in kidney transplant recipients is characterized by a predominance of *Enterobacterales* and mainly uropathogenic *Escherichia coli* (UPEC), *Enterococci*, and *Staphylococci* with a notable incidence of antibiotic resistance [4,5]. This resistance complicates the management of UTIs, emphasizing the importance of selecting appropriate antimicrobial therapy and considering the impact of antibiotic resistance patterns in this vulnerable population.

Diabetes mellitus has been identified as a significant risk factor for the development of UTIs. This association is linked to the impaired immune response and alterations in the urinary tract environment due to hyperglycemia, which facilitates bacterial colonization and infections [5]. Numerous studies highlight UTIs as the most prevalent infection among individuals with diabetes. A recent meta-analysis showed a prevalence for UTIs of 11.5% in a cohort of 872,948 diabetic patients [6]. Another study, conducted by Nichols et al. in 2017, reported a UTI prevalence of 33.3% in a sample of 32,295 patients [7]. These findings indicate that both the prevalence and risk of UTIs are significantly elevated in all diabetic patients.

In kidney transplant recipients, diabetes has been reported as a risk factor for UTIs, but the distinction between pre-transplant diabetes and post-transplant diabetes mellitus (PTDM) has rarely been made. In addition, previous studies reporting UTIs have often analyzed all UTIs together, including asymptomatic bacteriuria and cystitis, leading to conflicting results regarding patient and graft outcomes. In this context, we sought to investigate the association between diabetes, both pre-existing diabetes mellitus and PTDM, and the occurrence of clinically significant UTIs (pyelonephritis) after kidney transplantation. The secondary aims were (1) to assess the risk factors for UTIs, (2) to evaluate the interaction between diabetes and the occurrence of UTIs on patient survival, and (3) to analyze the microbiologic spectrum of UTIs in relation to diabetes status.

## 2. Patients and Methods

### 2.1. Study Design

This retrospective cohort study included individuals aged 18 years and older who underwent kidney transplantation at our tertiary academic hospital, in Brussels, from 1 January 2018 to 31 December 2022. Patients who received multi-organ transplants were excluded. Patient demographics and clinical characteristics were extracted from electronic medical records.

Participants were divided into three distinct categories: those diagnosed with diabetes before transplantation, those with post-transplant diabetes mellitus (PTDM), and non-diabetic patients. PTDM was classically defined as fasting blood glucose ≥ 126 mg/dL on multiple occasions, blood glucose ≥ 200 mg/dL with symptoms of polyuria, polydipsia, or polyphagia, blood glucose ≥ 200 mg/dL two hours after an oral glucose tolerance test, or glycated hemoglobin (HbA1) ≥ 6.5%—in all cases, requiring treatment.

### 2.2. Immunosuppression and Management Post-Transplantation

Patients with low immunological risk—defined in our institution as a 0% virtual panel reactive antibody (vPRA)—received no induction therapy if transplanted with a kidney from a deceased donor and basiliximab on day 0 and day 4 (20 mg) if transplanted with a kidney from a living donor. For patients with vPRA > 0%, plasmapheresis was performed until 2020, and rabbit anti-thymocyte globulin (Thymoglobulin^®^) was administered thereafter. Maintenance immunosuppression included a combination of tacrolimus (Tac) (trough level range of 9–13 ng/mL during the first month after KT, 7–9 ng/mL in months 2 and 3, and 5–7 ng/mL thereafter), mycophenolic acid (MPA) (500 mg bid), and steroids (methylprednisolone 500 mg on day 0, 16 mg/day from D1 with reduction of 4 mg every two weeks to reach a daily dose of 4 mg/day, continued lifelong). A double J catheter was placed during transplantation at the discretion of the surgeon, and removed after three weeks. Prophylactic treatment, including co-trimoxazole (3 times/week) and valganciclovir (except in *Cytomegalovirus* [CMV] serostatus D−/R−), was given for 6 months. Kidney biopsy was performed in case of increased creatinine, proteinuria, hematuria, or emergence of de novo donor-specific antibody.

### 2.3. Urinary Tract Infections

We identified UTIs according to the definitions of the American Society of Transplantation guidelines [8]. Asymptomatic bacteriuria was defined as the presence of over 10^5^ bacterial colony-forming units per milliliter (CFU/mL) in the urine without accompanying urinary or systemic symptoms. Simple cystitis/uncomplicated UTIs (Lower Tract UTIs) were defined by significant bacterial growth in urine cultures (>10 White Blood Cells (WBC)/mm^3^ and >10^3^ CFU/mL of uropathogens) and symptoms including dysuria, pollakiuria, and urinary urgency, but without systemic manifestations such as fever, graft pain, or hemodynamic instability. Complicated UTIs (Upper Tract UTIs) were defined as pyelonephritis characterized by systemic symptoms (fever, chills, hemodynamic instability, leukocytosis, and bacteremia) and significant bacterial growth (>10 WBC/mm^3^ and >10^4^ CFU/mL of uropathogens). Recurrent UTI was defined as three or more UTIs within a 12-month period.

In our study, we included complicated and recurrent UTIs. Asymptomatic bacteriuria and uncomplicated lower UTIs were excluded.

### 2.4. Statistical Analysis

Statistical analysis was performed using R (version 4.3.2), R Core Team, Vienna, Austria, 2022, and STATA (version 15.0). Categorical variables were expressed as counts with percentages and continuous variables as mean (±SD) or median [25th–75th percentiles] as appropriate. Differences in characteristics between groups were assessed using the χ^2^ test or Fischer Exact test for categorical variables and ANOVA or Kruskal–Wallis test for continuous variables as appropriate. Kaplan–Meier curves were used to evaluate a first UTI occurrence (censored at death, graft loss, and date of last follow-up), patient survival (censored at date of last follow-up), and graft loss (censored at death or date of last follow-up). Differences between groups were computed using a pairwise Log-Rank test. All *p*-values were adjusted for multiple testing using Bonferroni when applicable. All baseline variables with <10% missing values were considered potential risk factors for univariable analysis. Multivariable proportional hazard Cox regression models were used to find predictors of UTIs. All variables with a *p*-value < 0.2 in univariate analysis were considered to build models, and the best model was selected using likelihood ratio tests. An interaction model was built to evaluate the effect of pre-transplant diabetes combined with a UTI on survival, and the effect of pre-transplant diabetes combined with a double J catheter at the time of transplantation on the incidence of UTI. All *p*-values were two-tailed, with a *p*-value of less than 0.05 considered significant.

### 2.5. Ethical Considerations

All data were collected and processed with full respect for confidentiality and medical ethics. The study adhered to the principles of the Declaration of Helsinki and was approved by the ethics committee of the Cliniques Universitaires Saint-Luc (Reference 2023/03AVR/166—Date of approval 3 April 2023).

## 3. Results

### 3.1. Flow Chart and Subgroups

**Figure 1:**
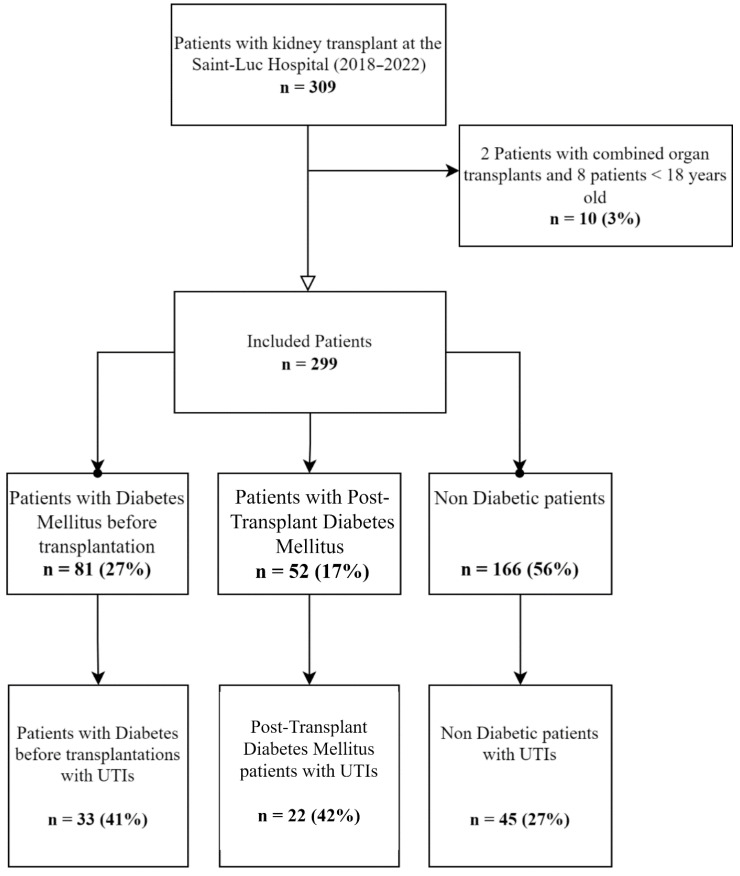
Flowchart of patient distribution according to diabetic status and UTIs occurrence.

We reviewed the files of three hundred and nine patients transplanted with a kidney from 2018 to 2022. After excluding pediatric patients and multi-organ transplants, we included 299 KTR. Patients were stratified according to their diabetic status prior to and following kidney transplantation. A total of 81 patients (27%) had pre-existing diabetes, 52 (17%) developed PTDM, and 166 (56%) remained non-diabetic. The mean time between transplantation and PTDM diagnosis was 12.3 ± 15 months. The occurrence of UTIs post-transplantation within each subgroup was 41% in patients with pre-transplant diabetes, 42% in patients with PTDM, and 27% in non-diabetic patients (Figure 1). The median (IQR) follow-up time was 29 (15–45) months.

### 3.2. Patient’s Characteristics

Demographics and patients’ characteristics in the three groups (pre-transplant diabetics, PTDM and non-diabetics) are detailed in Table 1. The mean age at transplantation was 52 ± 13 years. One-third of the included patients were women. Eighteen percent of KTR were transplanted for diabetic nephropathy. All patients received a triple maintenance immunosuppressive therapy consisting of Tac/MPA/steroids.

### 3.3. Occurrence of UTIs in Pre-Transplant Diabetics, PTDM, and Non-Diabetics

**Figure 2:**
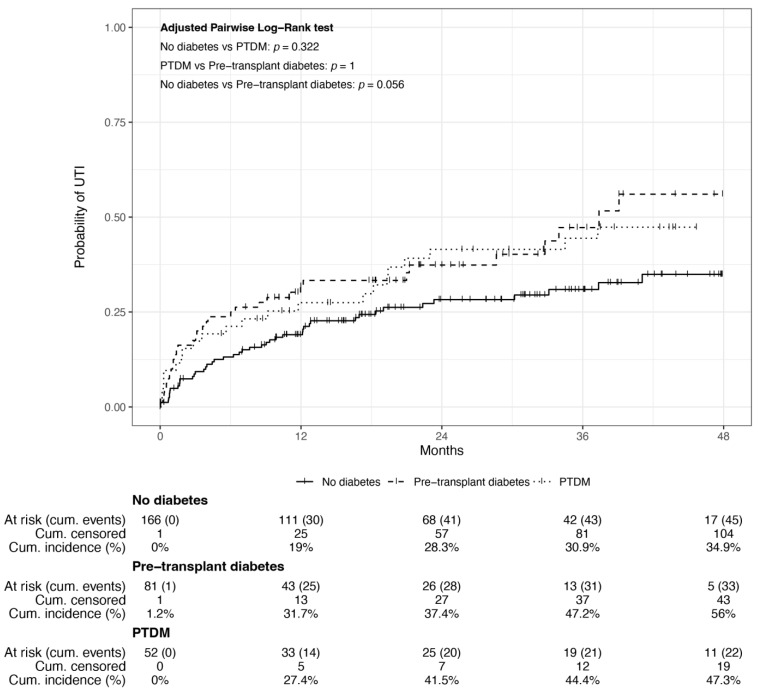
Cumulative incidence of urinary tract infection in pre-transplant diabetics, PTDM, and non-diabetics.

During a median follow-up time of 31 [17–45] months, 100 UTIs were reported in the total cohort, with a mean time from transplantation to the first UTI episode of 10 ± 11 months. The mean time between PTDM and UTI was 1.54 ± 9.6 months. At 48 months, the cumulative incidence of UTI was 34.9%, 56%, and 47.3% for patients without prior diabetes, pre-transplant diabetes, and PTDM, respectively (Figure 2). A trend towards higher UTI incidence was observed in patients with prior diabetes compared to those without prior diabetes to transplant (adjusted pairwise Log-rank test *p* = 0.056).

### 3.4. Risk Factors for UTIs

**Figure 3:**
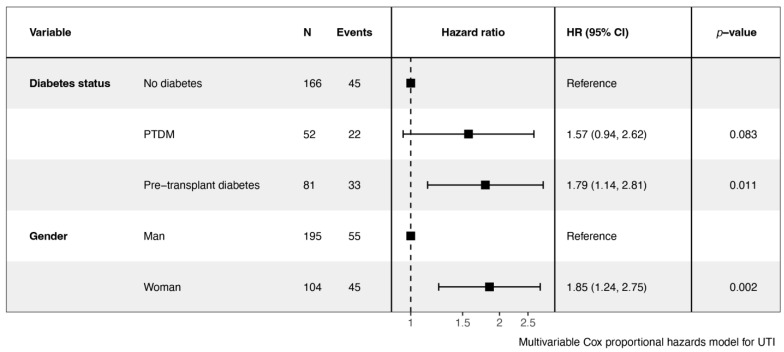
Multivariable Cox proportional hazards regression analysis, adjusting for potential confounders to identify independent predictors of UTIs. Hazard ratios are graphically represented with a square with their confidence interval. The dashed line refers to the reference (hazard ratio = 1). PTDM = Post-Transplant Diabetes Mellitus, UTI = Urinary Tract Infection, N = Number of patients, HR = Hazard Ratio.

Risk factors for UTIs among KTR were identified in a univariable Cox proportional hazards regression analysis (Table 2). Notably, pre-transplant diabetes was associated with an increased risk of UTIs (Hazard Ratio [HR] = 1.69, 95% Confidence Interval [CI] = 1.08–2.64, *p* = 0.023). A substantial increase in UTI risk was noted in female recipients (HR = 1.76, 95% CI = 1.19–2.61, *p* = 0.005). Other variables, including post-transplant diabetes, age, BMI, obesity, time on dialysis, induction treatment, and JJ catheterization did not show significant associations. A multivariable Cox proportional hazards regression analysis was built to identify independent predictors of UTIs (Figure 3). Pre-transplant diabetes was independently associated with 79% increased risk of UTIs (adjusted HR = 1.79, 95% CI = 1.14–2.81, *p* = 0.011). The risk associated with the female gender increased to 85% (adjusted HR = 1.85, 95% CI = 1.24–2.75, *p* = 0.002). An interaction model revealed no increased risk of UTI in patients with pre-transplant diabetes and double J catheter at transplantation (*p*[interaction] = 0.4).

### 3.5. Microbiological Spectrum of UTIs

**Figure 4:**
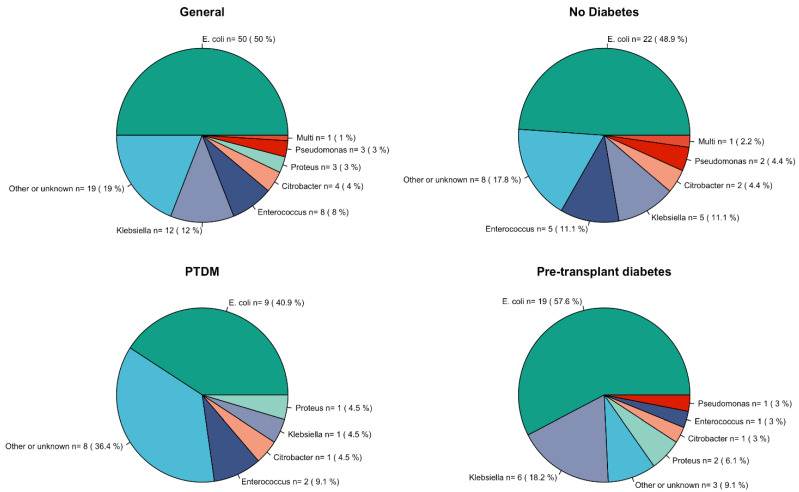
Microorganisms distribution in different groups.

*Escherichia coli* was the predominant pathogen, detected in 50 patients (50%), with the highest prevalence among pre-transplant diabetics (57.6%). *Enterococcus faecalis* was identified in eight patients, with a slightly higher occurrence in non-diabetics and PTDM patients. *Klebsiella pneumoniae* accounted for 12% of infections, predominantly in pre-transplant diabetics and non-diabetics. *Proteus mirabilis* and *Citrobacter freundii* were also present in different patients, showing variable distribution across diabetic and non-diabetic groups (Figure 4). The mean duration of antibiotic therapy for the total cohort was 13 ± 6 days. Recurrent UTIs occurred in 16% of the cohort.

### 3.6. Patient and Graft Outcomes During Follow-Up

During the follow-up, 28 KTR died (13 among the pre-transplant diabetics, 2 among the PTDM, and 13 among the non-diabetics), with a median time between transplantation and death of 19.5 [10.5–30] months. Causes of death were mostly related to infections (60% in pre-transplant diabetics vs. 33% in non-diabetics), while cardiovascular and neoplastic causes were similar in pre-transplant diabetics and non-diabetics (20% vs. 26.6% and 10% vs. 7%, respectively). Although patient survival was worst in pre-transplant diabetics, it was not significant compared to non-diabetics (adjusted pairwise log-rank test *p* = 0.143). Pre-transplant diabetic patients with UTIs had no increased risk of mortality as shown by an interaction model between diabetes and UTI to predict mortality (*p*[interaction] = 0.7).

Eighteen kidney grafts were lost during the follow-up and cumulative incidence at 48 months was similar in all groups (6 (7.4%) among the pre-transplant diabetics, 1 (1.9%) in the PTDM, and 11 (6.62%) among the non-diabetics, all adjusted pairwise log-rank test *p* > 0.2). Causes of graft loss in pre-transplant diabetics vs. non-diabetics were: primary non-function 33% vs. 18%, vascular 17% vs. 9%, unknown 33% vs. 18%, and non-adherence 17% vs. 18%.

## 4. Discussion

In this study, we evaluated the impact of diabetes, both pre-transplant and PTDM, on the occurrence of clinically significant UTIs in kidney transplant recipients. Our findings indicate that patients with pre-transplant diabetes and PTDM demonstrate a tendency toward increased incidence of UTIs. Pre-transplant diabetes emerged as a significant and independent risk factor for UTIs, in addition to female gender. There was no increased risk of UTI in pre-transplant diabetic patients who had a double J catheter placed during the transplant procedure. Patient survival was not significantly affected by the interaction between diabetes and UTI occurrence. Analysis of the microbiological spectrum of UTIs revealed the predominance of *E.coli* in all groups.

A higher occurrence of UTIs in KTR with diabetes mellitus corroborates with the existing scientific literature, which suggests that diabetes is linked to adverse outcomes, including an increased occurrence of UTIs [9]. As in our study, Ozawa et al. identified diabetes as a predictive risk factor for UTIs in a cohort of 236 KTR without specifying if diabetes was present before transplantation or developed as PTDM. Proposed mechanisms to explain this observation are that diabetic nephropathy leads to glucosuria, increased protein excretion, autonomic neuropathy, and immune system dysfunction [10]. Glucosuria as a result of poor diabetes control and bladder dysfunction leading to urine retention as a result of diabetic autonomic neuropathy creates a favorable environment for bacterial growth and proliferation. In addition, increased adherence of bacterial strains to the uroepithelial cells has been observed in patients with diabetes, particularly in those with poorly controlled diabetes [11]. Hyperglycemia also induces an immune dysfunction, affecting leukocyte function, adhesion, chemotaxis, and phagocytosis, and impairing cytokine secretion, leading to an inadequate immune response. Interestingly, hyperglycemia also induces dysbiosis of the gut microbiota, which may additionally influence susceptibility to UTIs [12].

Interestingly, we made a distinction between diabetes occurring before and after transplantation. We found that pre-transplant diabetes was significantly associated with the occurrence of UTIs with an HR of 1.79 (95% CI: 1.14–2.81), whereas PTDM did not reach significance. This lack of significant association for patients with PTDM could be attributed to the relatively short follow-up time. Recent findings [13] reported no significant differences in UTI occurrences among diabetic patients based on whether the duration of their diabetes was more or less than ten years, possibly explained by the progressive improvement in diabetes management over time leading to a decreased risk of UTI occurrences.

In addition to diabetes, our study highlights the traditional risk factors of the female gender as a significant predictor of UTIs. Indeed, it is well recognized that, because of their shorter urethra and its proximity to the anal and genital areas, women are more likely than men to develop UTIs. Moreover, the development of hormonal disturbances, especially the loss of estrogen, associated with the peri- and post-menopausal periods, contributes to increasing the risk of UTIs. This may influence our population whose mean age at transplantation was 50 [14]. Among the risk factors for UTIs, time on dialysis could be associated with an increased risk of UTIs, as prolonged dialysis can lead to bladder hypotrophy and reduced urinary capacity. In our cohort, time on dialysis was not found to affect the occurrence of UTIs. Another potential risk factor for UTIs is the double-J catheter inserted during the transplantation procedure. Classically, these ureteral stents are placed to protect the ureterovesical anastomosis, facilitate urine flow, and prevent obstruction [15]. Some groups advocate for the routine use of double-J catheter, but others have concerns that their use is associated with complications such as irritative voiding symptoms, hematuria, pain, migration occlusion, and UTIs [16]. Several mechanisms may explain the increased risk of UTI associated with ureteral stenting, namely traumatic injury to the ureter during insertion and the formation of a biofilm on the catheter surface. The length of time the catheter is left in place after implantation appears to influence the risk of occurrence of UTI [8]. Due to glucosuria and immune system dysfunction, diabetic patients may be more susceptible to foreign body infections. In kidney transplant recipients, the association between the use of double-J catheters and an increased risk of UTIs is controversial. Indeed, while some studies have reported an increased rate of UTI [13,15], others [17,18]—like ours—have not found a significant association between the two events. In our cohort, the use of double-J catheter was not associated with an increased risk of UTI in all patients including those with pre-transplant diabetes. Of note, our surgeons place the double-J catheter when deemed necessary, and the double-J catheter is removed no later than 3 weeks after insertion. This short insertion time likely contributes to reduce the risk of UTIs.

Consistent with reported studies [19], we observed a trend to increased mortality in pre-transplant diabetic patients compared with non-diabetics, but it did not reach significance. Interestingly, patients with PTDM had the best survival rates. Dos Santos et al. showed decreased patient survival in a cohort of 479 diabetic KTR. They suggested the cause could be due to the stimulation of the insulin-like growth factor axis affecting tumor development and the enhanced synthesis of pro-inflammatory cytokines, coupled with poor glycemic control in these patients [20]. Scheffner et al. [21] further demonstrated a significant increase in mortality and graft loss rates among diabetic patients experiencing UTIs. Similarly, Yeh et al. [22] found comparable results regarding patient survival. Their findings pointed to complications from severe infections leading to systemic sepsis as contributing factors to mortality. These observations could correlate with the occurrence of complicated UTIs among diabetic KTR and their detrimental impact on survival, emphasizing the need for vigilant infection monitoring and comprehensive diabetes care. In our cohort, an interaction model that included the effect of UTIs and pre-transplant diabetes on mortality did not show a significant effect on patient survival.

The existing literature highlights an increased risk of graft failure in diabetic KTRs. A meta-analysis by Lin et al. [23] reported a 35% higher risk of graft failure in diabetic KTRs compared to non-diabetic controls. The elevated risk in diabetic patients is attributed to factors such as acute rejection episodes, opportunistic infections, particularly within the first year post-transplant, and the aggressive use of immunosuppressive therapy, which can exacerbate hyperglycemia and its complications. In contrast, in our cohort, we did not find significant differences in graft survival rates between diabetic and non-diabetic patients. A possible explanation could rely in the short follow-up time post-transplantation.

Analysis of microorganisms causing UTIs showed that *E. coli* was the most frequently isolated pathogen across all categories, accounting for half of cases in total. The prevalence is slightly higher among pre-transplant diabetics (57.6%) compared to non-diabetics (48.9%). This supports the significance of *E. coli* as a dominant pathogen in UTIs, particularly among diabetic patients. Other pathogens such as *E. faecalis*, *K. pneumoniae*, *P. mirabilis*, and *C. freundii* are present in relatively smaller proportions. The distribution of these microorganisms suggests a diversity in the bacterial flora involved in UTIs among KTR. Similarly, Kamei et al. [24] showed that the microorganisms isolated from diabetic transplant patients are similar to those from non-diabetic patients, and that Gram-negative bacteria, including *E. coli*, are among the most prevalent pathogens in KTR.

We acknowledge the limitations of our study. Firstly, the retrospective nature of the research, conducted in a single center, introduces inherent biases related to the collection, availability, and accuracy of data, and the potential presence of unidentified confounding variables. Some potentially influential variables, such as the length of stay in the hospital and the surgical complications were not recorded. Secondly, the sample size and its specificity limit the ability to generalize the results to a broader patient population. In addition, the duration of follow-up is relatively short. Nevertheless, unlike many previous studies, our study has the advantage of including only clinically relevant UTIs while excluding simple cystitis, and our cohort has a close and accurate follow-up with detailed information on clinical, biological, microbiological and therapeutic handling of complications occurring after transplantation.

In conclusion, pre-transplant diabetes emerged as an independent risk factor for clinically significant UTIs in kidney transplant recipients, along with female gender. These findings advocate for integrated clinical strategies that not only target the prevention and treatment of UTIs but also address the broader complex metabolic challenges faced by kidney transplant recipients.

## Figures and Tables

**Table 1:** Patients characteristics at transplantation.

Characteristic	Overall, N = 299 ^1^	No Diabetes, N = 166 ^1^	Pre-Transplant Diabetes, N = 81 ^1^	PTDM, N = 52 ^1^
Recipient Age at Transplant	52 (13)	49 (14)	55 (12)	53 (10)
Gender				
Man	195 (65%)	106 (64%)	54 (67%)	35 (67%)
Woman	104 (35%)	60 (36%)	27 (33%)	17 (33%)
BMI	26.0 (4.9)	25.6 (4.5)	26.7 (5.4)	25.9 (5.0)
Nephropathy				
Glomerulonephritis/vasculitis	62 (22%)	49 (32%)	4 (5.1%)	9 (18%)
Diabetic nephropathy	49 (18%)	0 (0%)	49 (63%)	0 (0%)
Hypertensive large vessel disease	12 (4.3%)	5 (3.3%)	2 (2.6%)	5 (10%)
Interstitial nephritis	31 (11%)	20 (13%)	5 (6.4%)	6 (12%)
ADPKD	36 (13%)	23 (15%)	3 (3.8%)	10 (20%)
Etiology unknown or missing	78 (28%)	48 (31%)	15 (19%)	15 (31%)
Other	12 (4.3%)	8 (5.2%)	0 (0%)	4 (8.2%)
Dialysis Type				
Hemodialysis	266 (89%)	147 (89%)	71 (88%)	48 (92%)
Peritoneal dialysis	27 (9.0%)	15 (9.0%)	9 (11%)	3 (5.8%)
Preemptive	6 (2.0%)	4 (2.4%)	1 (1.2%)	1 (1.9%)
Time on Dialysis (Months)	51 (57)	49 (37)	54 (92)	53 (40)
Induction treatment (Yes)	84 (28%)	47 (28%)	23 (28%)	14 (27%)
Induction treatment (Type)				
No induction	215 (72%)	119 (72%)	58 (72%)	38 (73%)
Basiliximab	38 (13%)	21 (13%)	11 (14%)	6 (12%)
Rituximab	2 (0.7%)	2 (1.2%)	0 (0%)	0 (0%)
Plasmapheresis	44 (15%)	24 (14%)	12 (15%)	8 (15%)
Kidney Transplant Rank				
1	257 (88%)	139 (86%)	73 (92%)	45 (88%)
2	32 (11%)	20 (12%)	6 (7.6%)	6 (12%)
3	3 (1.0%)	3 (1.9%)	0 (0%)	0 (0%)
Donor Type				
Deceased	254 (85%)	141 (85%)	69 (85%)	44 (85%)
Living	45 (15%)	25 (15%)	12 (15%)	8 (15%)
Maintenance Triple Therapy	280 (94%)	158 (95%)	74 (91%)	48 (92%)
JJ catheterization at transplantation	192 (64%)	120 (72%)	40 (50%)	32 (62%)

^1^ Mean (SD); n (%).

**Table 2:** Univariable Cox regression analysis of risk factors for UTIs.

Univariable Cox Regression for UTI	N	HR ^1^	95% CI ^1^	*p*-Value
Diabetes Status	299			
No diabetes		—	—	
PTDM		1.51	0.91, 2.52	0.11
Pre-transplant diabetes		1.69	1.08, 2.64	**0.023**
Recipient Age at Transplant	297	1.01	0.99, 1.02	0.3
Gender	299			
Man		—	—	
Woman		1.76	1.19, 2.61	**0.005**
BMI	289	1.00	0.96, 1.04	0.9
Obesity BMI ≥ 30	299	0.98	0.60, 1.58	0.9
JJ catheterization at transplantation	298	0.79	0.53, 1.17	0.2
Dialysis Type	299			
Hemodialysis		—	—	
Peritoneal dialysis		0.69	0.32, 1.49	0.3
Preemptive		1.35	0.33, 5.50	0.7
Time on Dialysis (Months)	298	1.00	1.00, 1.00	0.9
Donor Type	299			
Deceased		—	—	
Living		1.12	0.66, 1.88	0.7
Transplant Rank (second or third)	292	1.43	0.78, 2.62	0.2
Induction treatment (Yes)	299	1.17	0.77, 1.78	0.5

^1^ HR, Hazard Ratio; CI, Confidence Interval. Bold values refer to statistically significant *p*-values (>0.05). PTDM = Post-Transplant Diabetes Mellitus, BMI = Body Mass Index.

## Data Availability

The raw data supporting the conclusions of this article will be made available by the authors on request.

## References

[1] Masci J.R., Wormser G.P. (2005). Mandell, Douglas, Bennett’s Principles and Practice of Infectious Diseases.

[2] Hosseinpour M., Pezeshgi A., Mahdiabadi M.Z., Sabzghabaei F., Hajishah H., Mahdavynia S. (2023). Prevalence and risk factors of urinary tract infection in kidney recipients: A Meta-analysis study. BMC Nephrol..

[3] Fiorentino M., Pesce F., Schena A., Simone S., Castellano G., Gesualdo L. (2019). Updates on urinary tract infections in kidney transplantation. J. Nephrol..

[4] Saliba W., Nitzan O., Chazan B., Elias M. (2015). Urinary tract infections in patients with type 2 diabetes mellitus: Review of prevalence, diagnosis, and management. Diabetes Metab. Syndr. Obes. Targets Ther..

[5] Bodro M., Sanclemente G., Lipperheide I., Allali M., Marco F., Bosch J., Cofan F., Ricart M., Esforzado N., Oppenheimer F. (2015). Impact of Antibiotic Resistance on the Development of Recurrent and Relapsing Symptomatic Urinary Tract Infection in Kidney Recipients. Am. J. Transplant..

[6] Salari N., Karami M.M., Bokaee S., Chaleshgar M., Shohaimi S., Akbari H., Mohammadi M. (2022). The Prevalence of Urinary Tract Infections in Type 2 Diabetic Patients: A Systematic Review and Meta-Analysis. Eur. J. Med. Res..

[7] Nichols G.A., Brodovicz K.G., Kimes T.M., Déruaz-Luyet A., Bartels D.B. (2017). Prevalence and incidence of urinary tract and genital infections among patients with and without type 2 diabetes. J. Diabetes Its Complicat..

[8] Goldman J.D., Julian K. (2019). Urinary tract infections in solid organ transplant recipients: Guidelines from the American Society of Transplantation Infectious Diseases Community of Practice. Clin. Transplant..

[9] Cantarin M.P.M. (2021). Diabetes in Kidney Transplantation. Adv. Chronic Kidney Dis..

[10] Ozawa K., Takai M., Taniguchi T., Kawase M., Takeuchi S., Kawase K., Kato D., Iinuma K., Nakane K., Koie T. (2022). Diabetes Mellitus as a Predictive Factor for Urinary Tract Infection for Patients Treated with Kidney Transplantation. Medicina.

[11] Geerlings S., Fonseca V., Castro-Diaz D., List D., Parikh S. (2014). Genital and urinary tract infections in diabetes: Impact of pharmacologically-induced glucosuria. Diabetes Res. Clin. Pract..

[12] Worby C.J., Schreiber H.L., Straub T.J., van Dijk L.R., Bronson R.A., Olson B.S., Pinkner J.S., Obernuefemann C.L.P., Muñoz V.L., Paharik A.E. (2022). Longitudinal multi-omics analyses link gut microbiome dysbiosis with recurrent urinary tract infections in women. Nat. Microbiol..

[13] Boyko E.J., Fihn S.D., Scholes D., Chen C.-L., Normand E.H., Yarbro P. (2002). Diabetes and the Risk of Acute Urinary Tract Infection Among Postmenopausal Women. Diabetes Care.

[14] Caretto M., Giannini A., Russo E., Simoncini T. (2017). Preventing urinary tract infections after menopause without antibiotics. Maturitas.

[15] Jonas M., Jóźwik A., Kawecki D., Durlik M., Pączek L., Młynarczyk G., Chmura A. (2016). Influence of Double-J Catheters on Urinary Infections After Kidney Transplantation. Transplant. Proc..

[16] Mosqueda A.O., Hernández E.E.L., Morales G.C., Navarro L.J.M., Bonilla J.P.H., Moreno E.O., Ugarte D.H. (2021). Association Between the Placement of a Double-J Catheter and the Risk of Urinary Tract Infection in Renal Transplantation Recipients: A Retrospective Cohort Study of 1038 Patients. Transplant. Proc..

[17] Dębska-Ślizień A., Bobkowska-Macuk A., Bzoma B., Moszkowska G., Milecka A., Zadrożny D., Wołyniec W., Chamienia A., Lichodziejewska-Niemierko M., Król E. (2018). Paired Analysis of Outcomes After Kidney Transplantation in Peritoneal and Hemodialysis Patients. Transplant. Proc..

[18] Velioglu A., Guneri G., Arikan H., Asicioglu E., Tigen E.T., Tanidir Y., Tinay I., Yegen C., Tuglular S. (2021). Incidence and risk factors for urinary tract infections in the first year after renal transplantation. PLoS ONE.

[19] Harding J.L., Pavkov M., Wang Z., Benoit S., Burrows N.R., Imperatore G., Albright A.L., Patzer R. (2021). Long-term mortality among kidney transplant recipients with and without diabetes: A nationwide cohort study in the USA. BMJ Open Diabetes Res. Care.

[20] Dos Santos Q., Hornum M., Terrones-Campos C., Crone C.G., Wareham N.E., Soeborg A., Rasmussen A., Gustafsson F., Perch M., Soerensen S.S. (2022). Posttransplantation Diabetes Mellitus Among Solid Organ Recipients in a Danish Cohort. Transpl. Int..

[21] Scheffner I.D., Gietzelt M., Abeling T., Marschollek M., Gwinner W. (2020). Patient Survival After Kidney Transplantation: Important Role of Graft-sustaining Factors as Determined by Predictive Modeling Using Random Survival Forest Analysis. Transplantation.

[22] Yeh H., Lin C., Li Y.R., Yen C.L., Lee C.C., Chen J.S., Chen K.H., Tian Y.C., Liu P.H., Hsiao C.C. (2020). Temporal trends of incident diabetes mellitus and subsequent outcomes in patients receiving kidney transplantation: A national cohort study in Taiwan. Diabetol. Metab. Syndr..

[23] Lin H., Yan J., Yuan L., Qi B., Zhang Z., Zhang W., Ma A., Ding F. (2021). Impact of diabetes mellitus developing after kidney transplantation on patient mortality and graft survival: A meta-analysis of adjusted data. Diabetol. Metab. Syndr..

[24] Kamei J., Yamamoto S. (2021). Complicated urinary tract infections with diabetes mellitus. J. Infect. Chemother..

